# 603. Comparing assays for Clinical Phage Microbiology in biofilm

**DOI:** 10.1093/ofid/ofac492.655

**Published:** 2022-12-15

**Authors:** Amit Rimon, Shunit Coppenhagen, Ran Nir-Paz, Ronen Hazan

**Affiliations:** The Hebrew University, Nofit, Hefa, Israel; The Hebrew University, Nofit, Hefa, Israel; Hadassah-Hebrew University Medical Center, Jerusalem, Yerushalayim, Israel; The Hebrew University, Nofit, Hefa, Israel

## Abstract

**Background:**

Non-resolving infections are commonly associated with bacterial biofilm formation. One promising solution is the use of lytic bacterial bacteriophages, as an adjunctive approach with antibiotics to penetrate biofilms and cure infections.

A major challenge in the use of phages for therapy is the need for accurate personal matching of the most effective phage-antibiotic combination against the target bactera. This matching, termed “Clinical Phage Microbiology” (CPM) is even more problematic regarding biofilm and there are no standard methods for that. Here we compared several approaches for CPM in biofilms.

**Methods:**

The efficacy of phages on the biofilm was tested in 8 methods using 5 phages at two concentrations. To this end five *Pseudomonas aeruginosa (PA)* specific phages were grown for biofilm establishment. Next, we followed the metabolic activity of the cultures using: (1)Agilent Seahorse XF Analyzers., and (2) Symcel’s calScreener calorimeter. In addition, we measured the levels of extracellular DNA in the biofilm supernatant using (3) rtPCR and (4) online by SYBR Green without amplification. The 5^th^ method used GFP PA14 with an allure-red dye that allows the assessment of the adherent bacteria only. Last, we used the commonly used (6) Colony Forming Units (CFU) counts, (7) Crystal Violet (CV), and (8) Live/dead stain as our gold standard controls.

**Results:**

Out of these methods, the calScreener (Figure 1A-F), and Seahorse were able to differentiate between phages real-time. The calSreener also showed a clear difference between biofilm and planktonic growth curves. The Real-Time PCR and the Live dead stain methods have shown a significant differentiation at the treatment endpoint. The other methods, CFU, CV, Syber Green, and Allura red did not show a significant difference between the phages. It should be noted that phages biofilm ranking did not correlate with the planktonic one.

**Figure 1:**
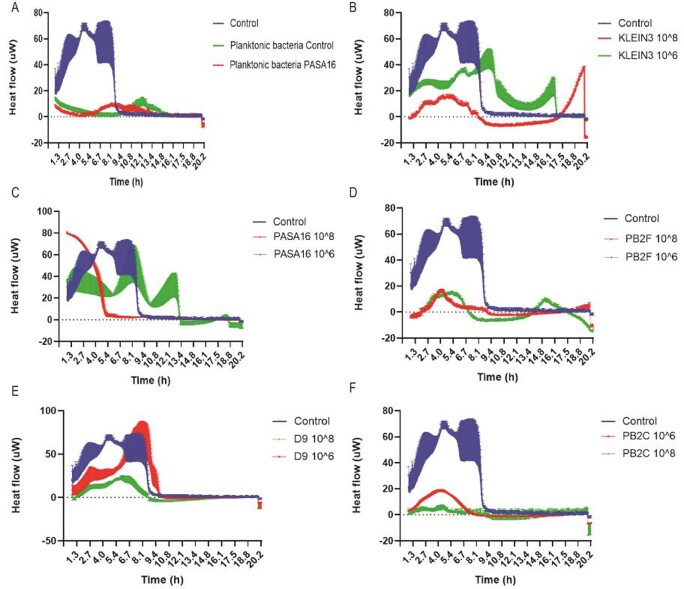

heat flow was measured using calScreener over 21 hours, comparing 5 different phages at a PFU of 108 and 106 on Pseudomonas aeruginosa planktonic (A) or biofilm (B-F). (A) planktonic bacteria and PASA16 phage treated, (B) KLEIN 3 phage, (C) PASA16 phage, (D) PB2F phage, (E) D9 phage, (F) PB2C phage.

**Figure 2:**
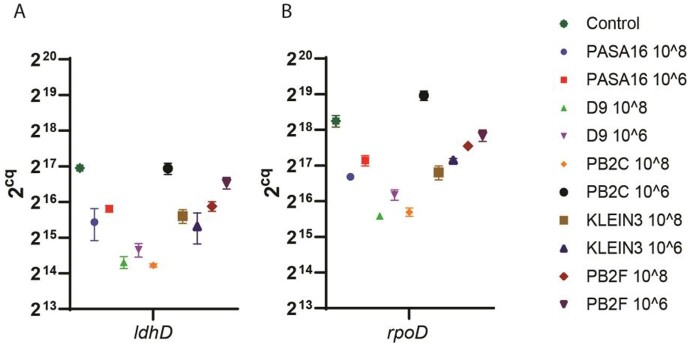

Comparison, using rtPCR, of 5 different phages at a PFU of 10^8 and 10^6 treated for 24 hours, and then their supernatant was filtered. Primers for housekeeping genes (A) ldhD, (B) rpoD.

**Conclusion:**

We have compared 8 different methods as assays for phage screening. In this model of PA biofilm and phages, we found that calScreener and Seahorse are superior while CFU, CV, Syber Green, Allura red are not as useful.

**Disclosures:**

**Ran Nir-Paz, MD**, BiomX: Advisor/Consultant|Technophage: Advisor/Consultant.

